# Impaired naming performance in temporal lobe epilepsy: language fMRI responses are modulated by disease characteristics

**DOI:** 10.1007/s00415-020-10116-x

**Published:** 2020-08-03

**Authors:** Karin Trimmel, Lorenzo Caciagli, Fenglai Xiao, Louis A. van Graan, Matthias J. Koepp, Pamela J. Thompson, John S. Duncan

**Affiliations:** 1 Epilepsy Society MRI Unit, Chalfont Centre for Epilepsy, Chalfont St Peter, SL9 0LR UK; 2 Department of Clinical and Experimental Epilepsy, UCL Queen Square Institute of Neurology, Queen Square, London, WC1N 3BG UK; 3 Department of Neurology, Medical University of Vienna, 1090 Vienna, Austria

**Keywords:** Language fMRI, Reorganisation, Temporal lobe epilepsy, Naming impairment, Disease characteristics, Deactivation

## Abstract

**Objective:**

To investigate alterations of language networks and their relation to impaired naming performance in temporal lobe epilepsy (TLE) using functional MRI.

**Methods:**

Seventy-two adult TLE patients (41 left) and 36 controls were studied with overt auditory and picture naming fMRI tasks to assess temporal lobe language areas, and a covert verbal fluency task to probe frontal lobe language regions. Correlation of fMRI activation with clinical naming scores, and alteration of language network patterns in relation to epilepsy duration, age at onset and seizure frequency, were investigated with whole-brain multiple regression analyses.

**Results:**

Auditory and picture naming fMRI activated the left posterior temporal lobe, and stronger activation correlated with better clinical naming scores. Verbal fluency MRI mainly activated frontal lobe regions. In left and right TLE, a later age of epilepsy onset related to stronger temporal lobe activations, while earlier age of onset was associated with impaired deactivation of extratemporal regions. In left TLE patients, longer disease duration and higher seizure frequency were associated with reduced deactivation. Frontal lobe language networks were unaffected by disease characteristics.

**Conclusions:**

While frontal lobe language regions appear spared, temporal lobe language areas are susceptible to dysfunction and reorganisation, particularly in left TLE. Early onset and long duration of epilepsy, and high seizure frequency, were associated with compromised activation and deactivation patterns of task-associated regions, which might account for impaired naming performance in individuals with TLE.

**Electronic supplementary material:**

The online version of this article (10.1007/s00415-020-10116-x) contains supplementary material, which is available to authorized users.

## Introduction

Temporal lobe epilepsy (TLE) is associated with impaired naming, particularly when seizure onset is lateralised to the speech-dominant hemisphere [1, 2]. Most clinically used paradigms in presurgical evaluation, such as verbal fluency or verb generation tasks, mainly activate frontal lobe language areas [3].

Temporal lobe language regions, especially posterior and basal temporal regions, are strongly involved in clinical naming performance [4], highlighting the importance of fMRI tasks that consistently activate these networks when evaluating TLE, such as the recently introduced auditory and visual naming fMRI paradigms [5]. Furthermore, successful task execution relies on the interplay of both task-positive and task-negative responses [6, 7], emphasizing the functional relevance of deactivation networks in language processing.

The underlying patterns of language reorganisation leading to naming impairment in TLE, particularly the role of temporal lobe language networks, as well as the effects of disease duration, age of onset and frequency of ongoing seizures are poorly understood.

We recently showed that psychophysiological interaction (PPI)-based functional connectivity of left posterior temporal lobe language networks was reduced with earlier age of onset of epilepsy and longer disease duration in left TLE [8]. The current study sample comprises our previously reported cohort [8] and an additional 11 participants, and builds on the findings of our previous work. Here, we focus on how naming fMRI networks are affected by disease characteristics of TLE, including the role of associated task-negative brain regions. We hypothesised that:

1. Task-positive and task-negative fMRI responses during naming and verbal fluency fMRI predict clinical naming performance
2. fMRI activation and deactivation patterns show greater perturbation in patients with early onset of seizures, long disease duration and high seizure burden.
3. Temporal lobe language networks are more affected by epilepsy-related reorganisation processes in TLE than are frontal lobe networks.

## Materials and methods

### Subjects

Seventy-two patients with drug-resistant TLE (36 females; age range 18–59 years; 41 left TLE) undergoing presurgical assessment at the National Hospital for Neurology and Neurosurgery (NHNN) between 2013 and 2017 were recruited. We also studied 36 healthy controls (20 females, age range 20–63 years; Table 1). Exclusion criteria were non-fluency in English, pregnancy, contraindication to MRI, inability to give informed consent, and history of a generalised tonic–clonic seizure within 24 h prior to the study.

**Table 1:** Demographic and clinical data for LTLE patients, RTLE patients and control subjects

	Gender female/male	Handedness right/left	Age (years)	Age onset (years)	Disease duration (years)	CPS monthly	SGS monthly	Number AED	Naming score	IQ	AN AccR (%)	PN AccR (%)
LTLE (*n* = 41)	20/21	37/4	36.6 ± 11.2	17.20 ± 8.0	14 (24)	4 (8)	0 (0)	2 (1.5)	14.6 ± 6.1^a^	96.0 ± 10.7^b^	90.0 (10.0)	97.8 (4.4)
RTLE (*n* = 31)	16/15	28/3	38.6 ± 10.4	21.4 ± 13.0	16 (15)	5 (8)	0 (0)	2 (1)	17.4 ± 5.2	98.9 ± 11.8^c^	90.0 (6.7)	97.8 (8.9)
CTR (*n* = 36)	20/16	33/3	37.6 ± 11.7	n.a	n.a	n.a	n.a	n.a	18.4 ± 5.5	108.8 ± 10.1	93.3 (10.0)	97.8 (4.4)

Age, age at onset of epilepsy, clinical naming scores and estimated intellectual level (IQ) are shown as mean ± SD. Disease duration, seizure frequency (CPS, SGS), in-scanner accuracy rates, and number of AED are shown as median and IQR

*AccR *in-scanner accuracy rate, *AED *antiepileptic drugs, *AN *auditory naming, *CPS *complex partial seizures, *CTR *control subjects, *IQ *estimated intellectual level, *IQR *interquartile range, *LTLE *left temporal lobe epilepsy, *PN *picture naming, *RTLE *right temporal lobe epilepsy, *SD *standard deviation, *SGS *secondarily generalised seizures

^a^Naming score LTLE < CTR *p* = 0.01

^b^IQ LTLE < CTR *p* < 0.001

^c^IQ RTLE < CTR *p* = 0.001

Prolonged EEG video telemetry confirmed and lateralised temporal seizure onset zones (ipsilateral in patients with structural brain lesions). All patients underwent structural MRI at 3 T, identifying hippocampal sclerosis (HS) in 34 patients (20 left/14 right), dysembryoplastic neuroepithelial tumour (DNET) in 13 (8 left/5 right), cavernoma in four (3 left/1 right), focal cortical dysplasia in two (1 left/1 right), low-grade glioma in three (1 left/2 right), dual pathology (FCD and HS) in one (right), encephalocoele in one (right), traumatic lesion in one (right) and normal-appearing MRI in 13 (8 left/5 right).

Handedness was determined using the Edinburgh Hand Preference Inventory [9]. The age distribution was comparable among the three groups (Table 1). The two patient groups did not differ for the age of onset of epilepsy, disease duration, seizure frequency or number of antiepileptic drugs (AEDs; Table 1). There is recent evidence that Topiramate and Zonisamide may affect language fMRI activation patterns [10, 11], however, there was no statistically significant difference between LTLE and RTLE in the number of patients treated with Topiramate (LTLE 1 patient, RTLE 2 patients, Fisher’s exact test: *p* = 0.40) or Zonisamide (LTLE 10 patients, RTLE 6 patients, Fisher’s exact test: *p* = 0.66) or the mean daily doses (Topiramate: Mann–Whitney *U* = 661, *p* = 0.40; Zonisamide: Mann–Whitney *U* = 599, *p* = 0.57).

### Neuropsychological tests

All subjects underwent neuropsychological testing prior to scanning. Naming was assessed using the McKenna Graded Naming Test, consisting of thirty line drawings of objects and animals, placed in order of difficulty [12]. Intellectual level was derived from performance on the National Adult Reading Test (NART, [13]).

### Statistical analysis

Statistical analyses were performed using SPSS 22.0 (Armonk, NY, USA). Group differences were explored with one-way analyses of variance (ANOVA). Correlations were performed with Pearson correlation coefficient *r* or Spearman’s rho*,* according to data distribution.

### MR data acquisition

MRI studies were performed using a 3T General Electric Signa MR750 scanner (GE, Wisconsin), using standard imaging gradients with a maximum strength of 50 mTm-1 and slew rate 200 TM-1 s-1. All data were acquired using the standard 32-channel RF receive head array coil and the body RF coil for transmission.

For fMRI, 50-slice gradient-echo planar T2*-weighted sequences, 24 cm field of view, TE = 22 ms, TR = 2500 ms, flip angle 90° were acquired, slice thickness 2.4 mm (0.1-mm gap), 64 × 64 matrix, giving an in-plane pixel size of 3.75 × 3.75 mm. The field of view was positioned to maximise coverage of the frontal and temporal lobes and minimise signal drop-out from the temporal and orbitofrontal lobes. The Array Spatial Sensitivity Encoding Technique (ASSET) was used to mitigate geometric distortions.

All subjects underwent a structural MRI scanning protocol on the same scanner, including an axial T1-weighted brain volume (BRAVO) sequence, a diffusion-weighted sequence, an axial and coronal T2-weighted sequence, an axial susceptibility-weighted sequence, and an oblique coronal 2D dual-echo proton density and T2-weighted image sequence.

### Language paradigms

We employed two overt language tasks, auditory naming, picture naming, and a covert verbal fluency paradigm, as described in detail previously [5, 8, 14].

Auditory naming consisted of five cycles of alternating 30-s activation blocks (naming aloud objects from the auditory description) alternating with 15-s control blocks of reversed speech (and overt response “one, two”) and cross-hair fixation (resting with eyes open).

Picture naming involved five cycles of alternating 30-s activation blocks (naming aloud line drawings of objects) and three 15-s control blocks of 15-s each, comprising scrambled pictures (SPc; overt response “one, two”), blurred cartoon faces (*F*; overt response “one, two”), and crosshair fixation (resting with eyes open).

Verbal fluency comprised 30-s activation blocks, requiring participants to covertly generate words beginning with a visually presented letter (A, S, W, D, E; one letter per block, 5 blocks in total), alternating with 30-s blocks of cross-hair fixation (resting with eyes open [3, 15].

All study participants successfully performed > 80% on the overt functional MRI tasks, without intergroup differences for both auditory naming (*H* = 5.619; *p* = 0.06) and picture naming (*H* = 1.038, *p* = 0.59). Due to technical problems with the audio and visual presentation systems, auditory naming could not be acquired in one LTLE patient and one control, and verbal fluency in two LTLE patients.

### fMRI data analysis

Imaging data were analysed using Statistical Parametric Mapping 8 (https://www.fil.ion.ucl.ac.uk/spm/). The imaging time series of each subject were realigned, normalised into standard anatomical space using a scanner- and acquisition-specific template (created from high-resolution whole brain echo planar images of 30 healthy controls, 15 patients with left HS, and 15 patients with right HS), and smoothed with a Gaussian kernel of 8 mm full-width at half-maximum.

A two-level random-effects analysis was employed. At the first level, condition-specific effects were estimated according to the general linear model [16]. Regressors of interest were formed by convolving blocks of stimuli with the canonical haemodynamic response function for each of the conditions of interest, using individual motion parameters as nuisance regressors. Parameter estimates for regressors were calculated voxel-wise. Three contrast images were generated for each subject, comprising (1) auditory naming minus reversed speech, (2) picture naming minus scrambled pictures and faces, and (3) verbal fluency.

At the second level, one-way ANOVA were used to quantitatively assess statistical differences among groups (LTLE, RTLE, controls). In the absence of significant intergroup differences, we investigated task effects across groups, using one-sample t-tests. Activations are reported at a threshold of *p* < 0.05, corrected for multiple comparisons (family-wise error rate [FWE]) across the whole brain.

#### Relation of fMRI activation to clinical naming performance and disease characteristics

The association of clinical naming performance, age of epilepsy onset, disease duration and seizure frequency with fMRI activation was explored with whole-brain multiple regression analyses [17]. Since the age of onset of epilepsy and disease duration were highly correlated (Spearman’s *ρ* = − 0.74; *p* < 0.001), we performed a supplementary analysis using the residuals of correlations with either age of onset or disease duration, in turn, as a covariate of no interest. Monthly frequency of focal seizures did not correlate with age at onset or disease duration. Group differences of correlations between groups were explored using *F*-contrasts in SPM.

In view of our a-priori hypothesis, all multiple regression activations are shown at an exploratory threshold of *p* < 0.001 uncorrected, in accord with previous investigations [8, 17]. To assess whether correlations were related to activation or deactivation, results were masked with binarized group activation and deactivation maps, respectively. Estimated verbal IQ derived from performance on the NART [13] was used as a covariate of no interest for all analyses.

## Results

### Neuropsychological language performance

Groups differed significantly with respect to estimated intellectual level (*F*(2,107) = 14.3; *p* < 0.001) and naming scores (*F*(2,107) = 4.9; *p* = 0.01). Post-hoc pairwise comparisons (Tukey HSD) indicated that mean estimated IQ was higher in controls than LTLE patients (*p* < 0.001) and RTLE patients (*p* = 0.001), while there was no significant difference between LTLE and RTLE patients (Table 1). LTLE patients performed significantly less well on the out-of-scanner naming task than controls (*p* = 0.01), while there was no difference in naming scores between LTLE and RTLE patients or between RTLE patients and controls (Table 1). Education level differed significantly between groups (*H*(2,108) = 15.298, *p* < 0.001), and post hoc pairwise comparison showed that controls had higher education level than LTLE (*p* = 0.001) and RTLE (*p* = 0.01), while there was no difference between LTLE and RTLE patients (*p* = 0.50). In LTLE patients, a later age at onset of epilepsy correlated with better clinical naming scores (Pearson’s *r* = 0.41; *p* = 0.01), which was not observed in RTLE patients (Pearson’s *r* = 0.20, p = 0.28). Naming scores did not significantly correlate with disease duration or seizure frequency in either patient group (disease duration: RTLE Spearman’s *ρ* = − 0.21, *p* = 0.26; LTLE Spearman’s *ρ* = 0.08, *p* = 0.63; seizure frequency: RTLE Spearman’s *ρ* = − 0.20, *p* = 0.28; LTLE Spearman’s *ρ* = 0.16, *p* = 0.32). Estimated verbal IQ correlated with better clinical naming scores (Pearson’s *r* = 0.45, *p* < 0.001), and there was a trend for higher IQ correlating with later age at onset (Pearson’s *r* = 0.22, *p* = 0.06). There was no correlation of IQ with disease duration (Spearman’s *ρ* = − 0.02, *p* = 0.89) or frequency of focal seizures (Spearman’s *ρ* = − 0.14, *p* = 0.23).

### fMRI results—main effects

#### Auditory naming

**Fig. 1:**
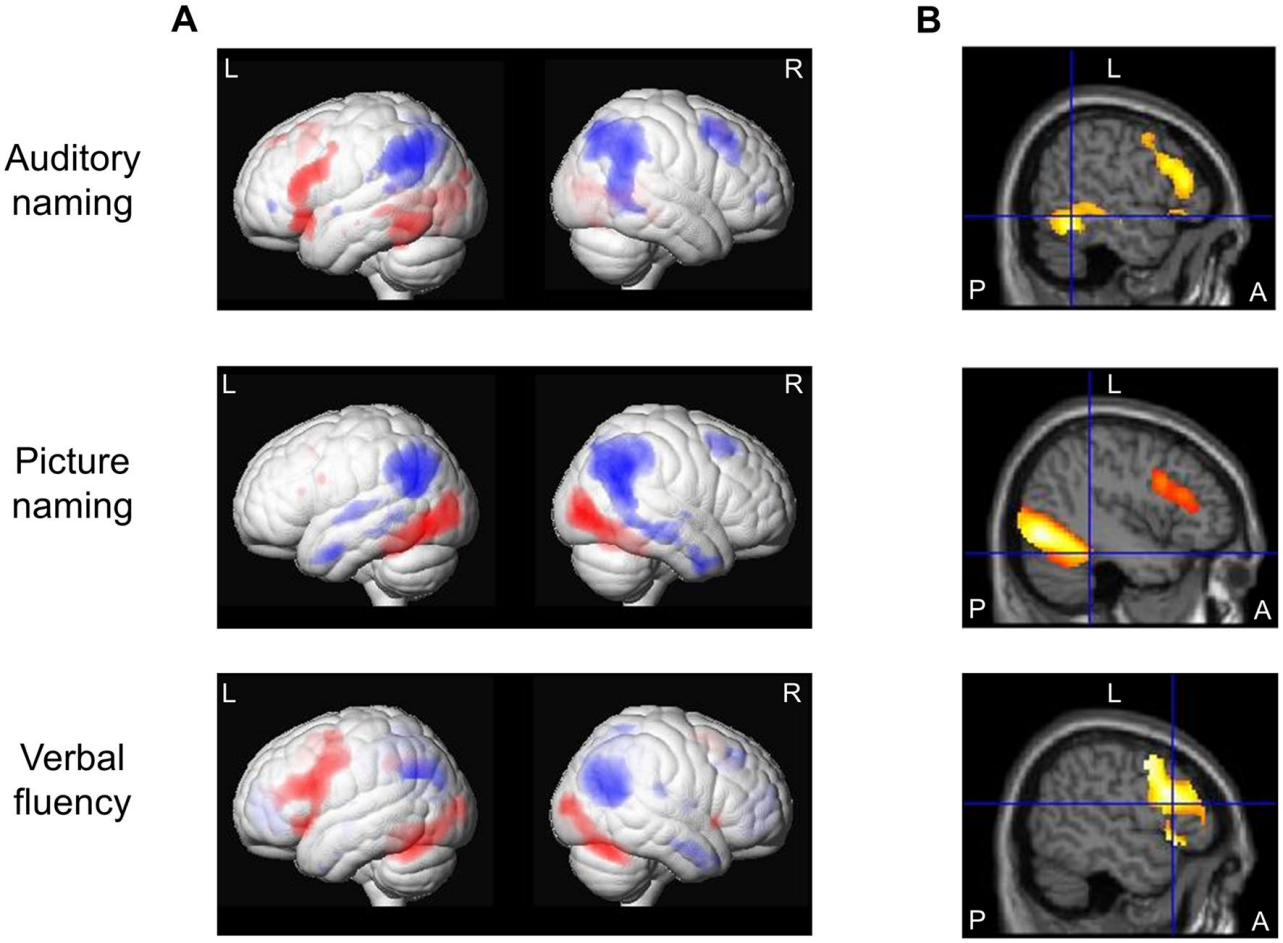
**a** Main fMRI activations (red) and deactivations (blue) across all three groups (left TLE, right TLE, controls) for auditory naming (upper row), picture naming (middle row) and verbal fluency (lower row) tasks shown rendered at *p* < 0.05, corrected for multiple comparisons (FWE). **b** Main fMRI activation across all three groups superimposed on sagittal images. Crosshairs indicate left inferior temporal gyrus fMRI activations for auditory naming, left fusiform gyrus activations for picture naming, and left inferior frontal gyrus activations for verbal fluency. Auditory naming: Sagittal slices also show left frontal activations. Picture naming: Sagittal slices also show left occipital and left frontal activations All activations are shown at *p* < 0.05, corrected for multiple comparisons (FWE). *A *anterior, *FWE *family-wise error, *L *left, *P *posterior, *R* right, *TLE* temporal lobe epilepsy

Task-related activations across groups were observed in the left posterior inferior temporal gyrus and anterior and posterior middle temporal gyrus, left temporal pole (superior temporal gyrus), left inferior and superior frontal gyrus and supplementary motor region, left anterior parahippocampal gyrus, left lingual gyrus, left superior occipital gyrus, and right thalamus (Fig. 1; Table 2).

**Table 2:** MNI coordinates and Z-scores of whole-brain cluster-level activations and deactivations across all subjects (left TLE, right TLE, controls) during auditory naming, picture naming and verbal fluency shown corrected for multiple comparisons (FWE; *p* < 0.05)

	Auditory naming				Picture naming				Verbal fluency			
	Left		Right		Left		Right		Left		Right	
	*Z*	Coordinates	*Z*	Coordinates	*Z*	Coordinates	*Z*	Coordinates	*Z*	Coordinates	*Z*	Coordinates
Whole-brain activations												
Inf front G	7.28	− 46 30 14			4.97	− 44 8 28			> 8	− 16 14 24	5.63	44 16 − 6
Sup front G	4.87	− 10 50 48										
Suppl motor	6.81	− 4 18 54			4.82	− 4 16 54			> 8	− 2 10 64		
Precentral G									> 8	− 54 0 44		
Inf temp G	7.65	− 48 − 52 − 18										
Mid temp G	5.12	− 52 − 34 − 4										
Sup temp G	6.59	− 42 24 − 18										
Parahipp G	4.47	− 20 − 20 − 22										
Fusiform G					7.45	− 34 − 46 − 24						
Lingual G	> 8	− 6 − 56 0										
Thalamus			4.64	20 − 18 18								
Inf occipit G					> 8	− 42 − 74 − 8	> 8	42 − 84 − 4				
Mid occip G					> 8	− 36 − 88 − 4			5.44	− 26 − 70 40	> 8	26 − 100 10
Sup occip G	4.49	− 24 − 80 34										
Cerebellum									7.75	− 36 − 66 − 26	> 8	36 − 66 − 28
Whole-brain deactivations												
Precuneus			> 8	4 − 74 38			> 8	2 − 68 42			> 8	4 − 64 32
Angular G	> 8	− 48 − 64 40	7.82	34 − 74 54	> 8	− 56 − 68 24	7.67	44 − 78 40	4.98	− 44 − 56 26		
Cingulate									> 8	− 10 − 48 34	> 8	6 − 52 32
Supramar G	> 8	− 62 − 48 38							> 8	− 56 − 66 32		
Inf par lob	5.69	− 62 − 56 18										
Mid front G	4.88	− 30 48 − 2	6.60	30 18 62			6.07	30 24 60			5.19	24 36 42
Sup front G			4.63	18 62 16								
Med front G											7.61	2 58 − 2
Precentral G			6.00	32 − 10 52								
Postcentr G											5.42	52 − 26 20
Temp pole										− 50 10 − 36		
Sup temp G	4.92	− 46 − 38 12			6.31	− 48 − 18 2						
Mid temp G					6.30	− 54 2 − 32			5.93	− 46 6 − 44	> 8	52 − 68 18
Inf temp G			4.45	58 − 62 − 14								
Fusiform G									5.02	− 28 − 34 − 20		
Parahipp G											4.43	24 − 18 − 30
Insula									4.50	− 44 − 16 10	4.92	34 4 10
Mid occip G									6.99	− 40 − 88 22	7.75	44 − 76 34

*Front *frontal, *G *gyrus, *inf *inferior, *L *left, *med *medial, *mid *middle, *MNI *Montreal Neurological Institute, *parahipp *parahippocampal, *postcentr *postcentral, *occip *occipital, *R *right, *supramar *supramarginal, *suppl motor *supplementary motor region, *temp *temporal, *TLE *temporal lobe epilepsy

Task-related deactivations across groups included the left supramarginal gyrus, left inferior parietal lobule, left anterior and posterior superior temporal gyrus, right precuneus, right inferior temporal gyrus, right superior frontal gyrus, right precentral gyrus as well as angular gyrus and middle frontal gyrus bilaterally (Fig. 1; Table 2). Intergroup comparisons did not reveal a significant difference in activation or deactivation patterns among groups.

#### Picture naming

Across groups, activations were seen in the left fusiform gyrus, left inferior frontal gyrus and the supplementary motor region as well as left middle and bilateral inferior occipital gyrus (Fig. 1; Table 2).

Task-related deactivations across groups were observed in the left anterior and posterior middle and superior temporal gyrus, the right precuneus, right middle frontal gyrus, and angular gyrus bilaterally (Fig. 1; Table 2). Group comparisons indicated no significant difference in activation or deactivation patterns among groups.

#### Verbal fluency

Main activations across groups were seen in the inferior frontal gyrus bilaterally (left > right), left precentral gyrus and left supplementary motor area, and middle occipital gyrus bilaterally, and cerebellum (right > left; Fig. 1; Table 2).

Task-related deactivations across groups were seen in the left angular gyrus, left supramarginal gyrus, left temporal pole, left fusiform gyus, right precuneus, right middle frontal gyrus, right medial frontal gyrus, right parahippocampal gyrus, right posterior middle temporal gyrus, right postcentral gyrus as well as in the bilateral cingulate, anterior middle temporal gyrus, insula, and middle occipital gyrus (Fig. 1; Table 2). Inter-group comparisons did not show significant differences in activation or deactivation patterns.

### Relation of fMRI activation to clinical naming performance

**Fig. 2:**
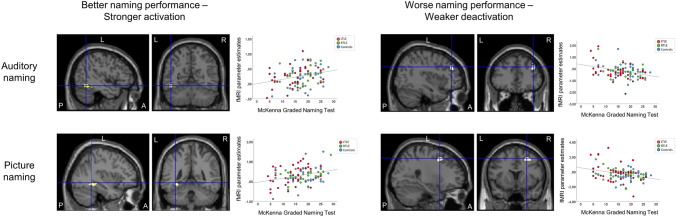
Relation of auditory naming and picture naming fMRI activations to out-of-scanner clinical naming performance across all three groups (left TLE, right TLE, controls). Activations are shown superimposed on coronal and sagittal images at *p* < 0.001, uncorrected and the crosshair indicates the orthogonal slices. Better clinical naming performance (left column) was associated with stronger activations in the left posterior inferior temporal gyrus during auditory naming (upper row), and in the left fusiform gyrus during picture naming (lower row). Worse clinical naming performance (right column) was related to weaker deactivation of the right superior frontal gyrus during both auditory naming and picture naming. Scatterplots show correlations of fMRI parameter estimate with naming scores (left TLE: red; right TLE: green; controls: blue). *A *anterior, *L *left, *P *posterior, *R *right, *TLE *temporal lobe epilepsy

Stronger fMRI activations in the left posterior inferior temporal gyrus during auditory naming, and in the left fusiform gyrus during picture naming, were associated with better clinical naming performance (Fig. 2; Table 3). Worse naming performance, on the other hand, related to reduced deactivation of the right middle frontal gyrus during both auditory naming and picture naming (Fig. 2; Table 3). Stronger verbal fluency activations in the left frontal lobe were associated with better naming scores (Table 3). These results were observed across all groups, without significant intergroup differences.

**Table 3:** MNI coordinates and *Z*-scores of correlations of cluster-level fMRI activation during auditory naming, picture naming, and verbal fluency with out-of-scanner naming scores across all groups, shown at *p* < 0.001 uncorrected

	Auditory naming				Picture naming				Verbal fluency			
	Left		Right		Left		Right		Left		Right	
	*Z*	Coordinates	*Z*	Coordinates	*Z*	Coordinates	*Z*	Coordinates	*Z*	Coordinates	*Z*	Coordinates
Better clinical naming performance												
All groups												
Inf temp G	3.42	− 46 − 56 − 14										
Fusiform G					3.18	− 34 − 50 − 14						
Cerebellum			3.49	32 − 66 − − 26								
Inf front G									3.24	− 38 12 44		
Worse clinical naming performance												
All groups												
Mid front G			3.13	40 36 38			3.64	28 12 50				

*Front *frontal, *G *gyrus, *inf *inferior, *mid *middle, *MNI *Montreal Neurological Institute

### Association of fMRI results with disease characteristics

#### Age at onset of epilepsy

**Fig. 3:**
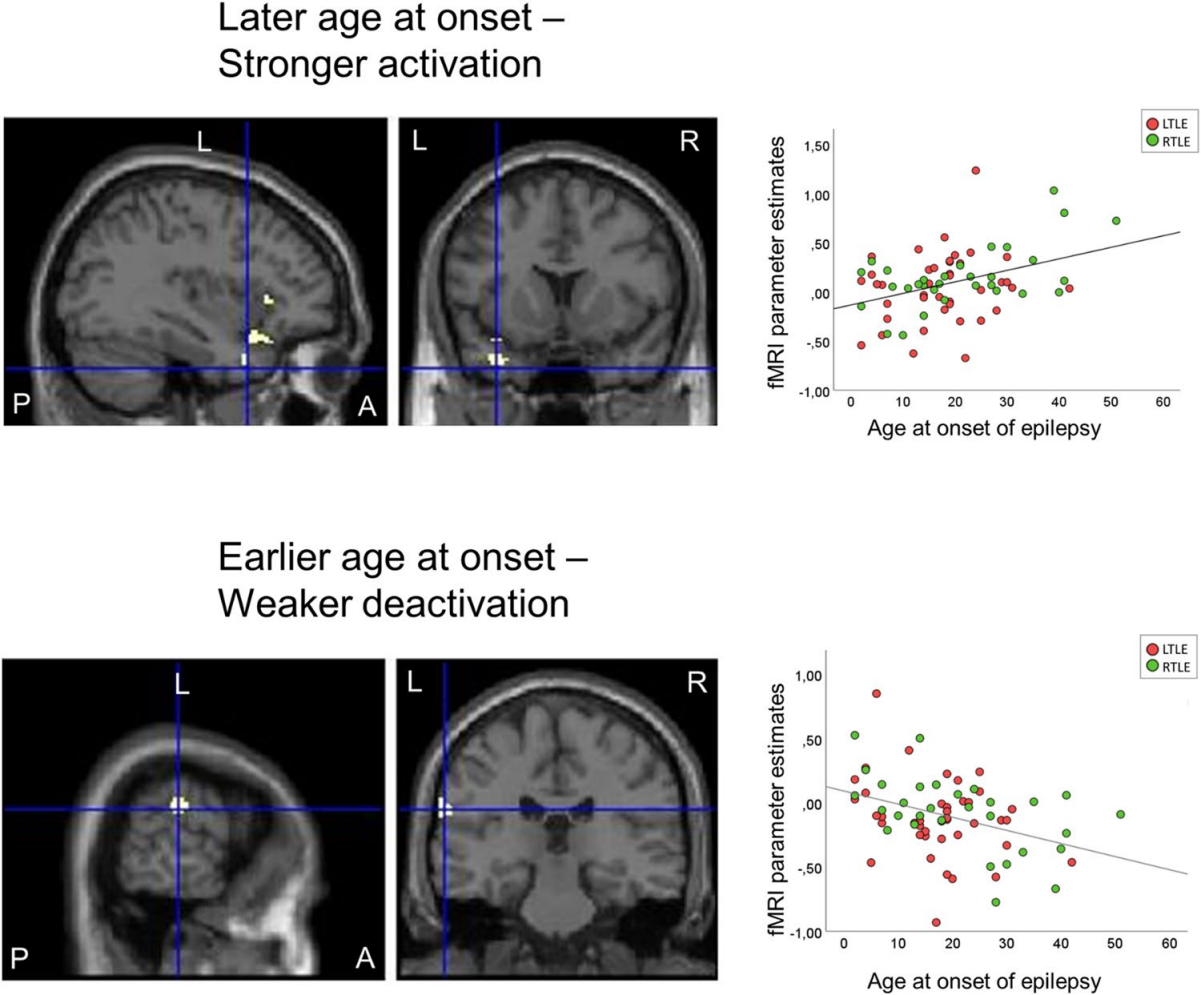
Relation of auditory naming fMRI activation to age at onset of epilepsy across LTLE and RTLE patients. Activations are shown superimposed on coronal and sagittal images at *p* < 0.001, uncorrected and the crosshair indicates the orthogonal slices. A later age of onset (top row) was associated with stronger activations in the left anterior superior and middle gyrus (crosshair) and left inferior frontal gyrus, whereas an earlier age of onset (lower row) was related to weaker deactivation of the left supramarginal gyrus. Scatterplots show correlations of fMRI parameter estimate with age at onset of epilepsy (left TLE: red; right TLE: green). *A *anterior, *L *left, *P *posterior, *R *right, *TLE *temporal lobe epilepsy

For auditory naming, later age at onset of epilepsy related to stronger activations in task-relevant areas, whereas earlier age of onset was associated with effects within deactivation networks across both patient groups, without intergroup differences. In detail, later age of onset of epilepsy was associated with stronger auditory naming activations in the left anterior middle temporal gyrus and the left inferior and middle frontal gyrus. An earlier age of epilepsy onset was associated with weaker deactivation of the left supramarginal gyrus during auditory naming, and of the right precuneus and right middle temporal gyrus during picture naming (Fig. 3; Table 4). Repeat analyses controlling for disease duration did not alter the main results (Table 5). A post-hoc analysis comparing patients with early onset of seizures (< 18 years, *n* = 33) and late onset (> 18 years, *n* = 39) showed that, at the whole-brain level, patients with early seizure onset showed less activation in the left temporal pole compared to controls (*p* < 0.05, FWE-corrected; Supplementary Fig. 1), while there was no difference between patients with later seizure onset compared to controls.

**Table 4:** MNI coordinates and *Z*-scores of correlations of cluster-level fMRI activation during auditory and picture naming with age at onset of epilepsy across left TLE and right TLE patients, shown at *p* < 0.001 uncorrected

	Auditory naming				Picture naming			
	Left		Right		Left		Right	
	*Z*	Coordinates	*Z*	Coordinates	*Z*	Coordinates	*Z*	Coordinates
Later age at onset								
LTLE + RTLE								
Sup temp G (ant)	3.64	− 34 10 − 32						
Mid temp G (ant)	3.33	− 54 − 12 − 22						
Inf front G	3.36	− 34 36 6						
Earlier age at onset								
LTLE + RTLE								
Supramarg G	3.25	− 66 − 26 26						
Precuneus							3.29	6 − 82 42

*Front *frontal, *G *gyrus, *inf *inferior, *LTLE *left temporal lobe epilepsy, *mid *middle, *MNI *Montreal Neurological Institute, *RTLE *right temporal lobe epilepsy, *supramarg *supramarginal, *temp *temporal

**Table 5:** MNI coordinates and *Z*-scores of correlations of cluster-level fMRI activation during auditory and picture naming with age at onset of epilepsy across LTLE and RTLE patients, shown at *p* < 0.001 uncorrected, using residuals of correlation with disease duration as covariate of no interest

	Auditory naming				Picture naming			
	Left		Right		Left		Right	
	*Z*	Coordinates	*Z*	Coordinates	*Z*	Coordinates	*Z*	Coordinates
Later age at onset								
LTLE + RTLE								
Mid temp G (ant)	3.37	− 54 − 12 − 22						
Inf front G	3.50	− 34 36 4						
Mid front G	3.74	− 54 22 32						
Earlier age at onset								
LTLE + RTLE								
Supramarg G	3.24	− 66 − 26 26						
Precuneus							3.29	6 − 82 52
Mid temp G							3.14	52 − 50 8

*Front *frontal, *G *gyrus, *inf *inferior, *LTLE *left temporal lobe epilepsy, *mid *middle, *MNI* Montreal Neurological Institute, *RTLE *right temporal lobe epilepsy, *supramarg *supramarginal, *temp* temporal

#### Disease duration

**Fig. 4:**
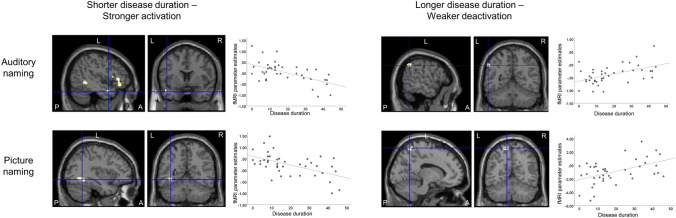
Relation of auditory naming and picture naming fMRI activations to disease duration in left TLE patients. Activations are shown superimposed on coronal and sagittal images at *p* < 0.001, uncorrected and the crosshair indicates the location on orthogonal slices. For auditory naming (upper row), shorter disease duration (left column) was associated with stronger activations in the left temporal pole (crosshair), left middle temporal gyrus, and left inferior frontal gyrus, whereas longer disease duration (right column) was related to weaker deactivation of the left inferior parietal lobule (crosshair). For picture naming (lower row), shorter disease duration (left column) was associated with stronger activations in the left fusiform gyrus (crosshair), and bilateral superior and middle occipital cortex (not shown on slice), whereas longer disease duration (right column) related to weaker deactivation of the left precuneus (crosshair) and right inferior parietal lobule (not shown on slice). Scatterplots show correlations of fMRI parameter estimate with disease duration. *A *anterior, *L *left, *P *posterior, *R *right. *TLE *temporal lobe epilepsy

For auditory naming, LTLE patients with shorter disease duration showed stronger activations in the left anterior middle temporal gyrus, left temporal pole (superior temporal gyrus) and left inferior frontal gyrus (Fig. 4; Table 6), part of the activation networks. Longer disease duration was related to weaker deactivation of the left inferior parietal lobule (Fig. 4; Table 6).

**Table 6:** MNI coordinates and *Z*-scores of correlations of cluster-level MRI activation during auditory and picture naming with disease duration and seizure frequency, shown at *p* < 0.001 uncorrected

	Auditory naming				Picture naming			
	Left		Right		Left		Right	
	*Z*	Coordinates	*Z*	Coordinates	*Z*	Coordinates	*Z*	Coordinates
Shorter disease duration								
LTLE								
Inf front G	4.22	− 48 30 6						
Mid temp G (ant)	3.11	− 58 0 − 18						
Sup temp G (pole)	3.88	− 44 8 − 26						
Fusiform G					3.31	− 36 − 46 − 16		
Mid occipit G					3.10	− 30 − 98 10		
LTLE > RTLE								
Inf front G	3.64	− 38 30 − 20						
Mid temp G (ant)	3.13	− 40 10 − 44						
Mid occipit G					3.74	− 48 − 74 − 10		
Fusiform G					3.43	− 40 − 60 − 14		
Longer disease duration								
LTLE								
Precuneus			3.28	4 − 54 48	3.43	− 4 − 66 52	3.06	4 − 64 60
LTLE > RTLE								
Supramarg G							3.16	32 − 52 36
Higher seizure frequency								
LTLE								
Angular G	3.97	− 44 − 56 34						
Inf par lob	3.42	− 42 − 30 36						
LTLE > RTLE								
Inf par lob	3.44	− 52 − 42 58						
Supramarg G	3.15	− 52 − 22 24						
Lower seizure frequency								
LTLE								
Fusiform G					3.53	− 38 − 46 − 18		
Inf occip G					3.29	− 46 − 84 − 8		
LTLE > RTLE								
Fusiform G					3.11	− 34 − 10 − 28		

*Front *frontal, *G *gyrus, *inf *inferior, *LTLE *left temporal lobe epilepsy, *lob *lobule, *mid *middle, *MNI *Montreal Neurological Institute, *occip *occipital, *RTLE *right temporal lobe epilepsy, *supramarg *supramarginal, *temp *temporal, *TLE *temporal lobe epilepsy

For picture naming, LTLE patients showed an association of shorter disease duration with stronger activations in the left fusiform gyrus and left middle occipital gyrus (Fig. 4; Table 6), part of the activation networks. Longer disease duration was related to weaker deactivation of the precuneus bilaterally and right supramarginal gyrus (Fig. 4; Table 6).

Group comparisons indicated stronger associations in LTLE compared to RTLE patients, who had no significant correlation with disease duration during auditory naming or picture naming. Supplementary analyses controlling for age at epilepsy onset did not alter the main results (Table 7).

**Table 7:** MNI coordinates and *Z-*scores of correlations of cluster-level fMRI activation during auditory and picture naming with disease duration across LTLE and RTLE patients, shown at *p* < 0.001 uncorrected, using residuals of correlation with age at onset of epilepsy as a covariate of no interest

	Auditory naming				Picture naming			
	Left		Right		Left		Right	
	Z	Coordinates	Z	Coordinates	Z	Coordinates	Z	Coordinates
Shorter disease duration								
LTLE								
Inf front G	4.34	− 48 30 4						
Mid front G	3.15	− 36 40 − 14						
Sup temp G (pole)	3.30	− 44 12 − 20						
Inf temp G (post)					3.11	− 46 − 68 − 4		
Fusiform G					3.52	− 36 − 54 − 8		
Mid occipit G					3.24	− 30 − 98 12		
LTLE > RTLE								
Inf front G	3.79	− 38 30 − 20						
Mid temp G (ant)	3.60	− 42 8 − 44						
Sup temp G (pole)	3.14	− 34 8 − 36						
Fusiform G					3.31	− 40 − 42 − 14		
Longer disease duration								
LTLE								
Precuneus			3.24	4 − 54 48	3.21	− 4 − 66 52		
LTLE > RTLE								
Supramarg G					3.24	32 − 52 36		

*Front *frontal, *G *gyrus, *inf *inferior, *LTLE *left temporal lobe epilepsy, *mid *middle, *MNI *Montreal Neurological Institute, *occip *occipital, *RTLE *right temporal lobe epilepsy, *sup *superior, *supramarg *supramarginal, *temp *temporal, *TLE *temporal lobe epilepsy

#### Seizure frequency

**Fig. 5:**
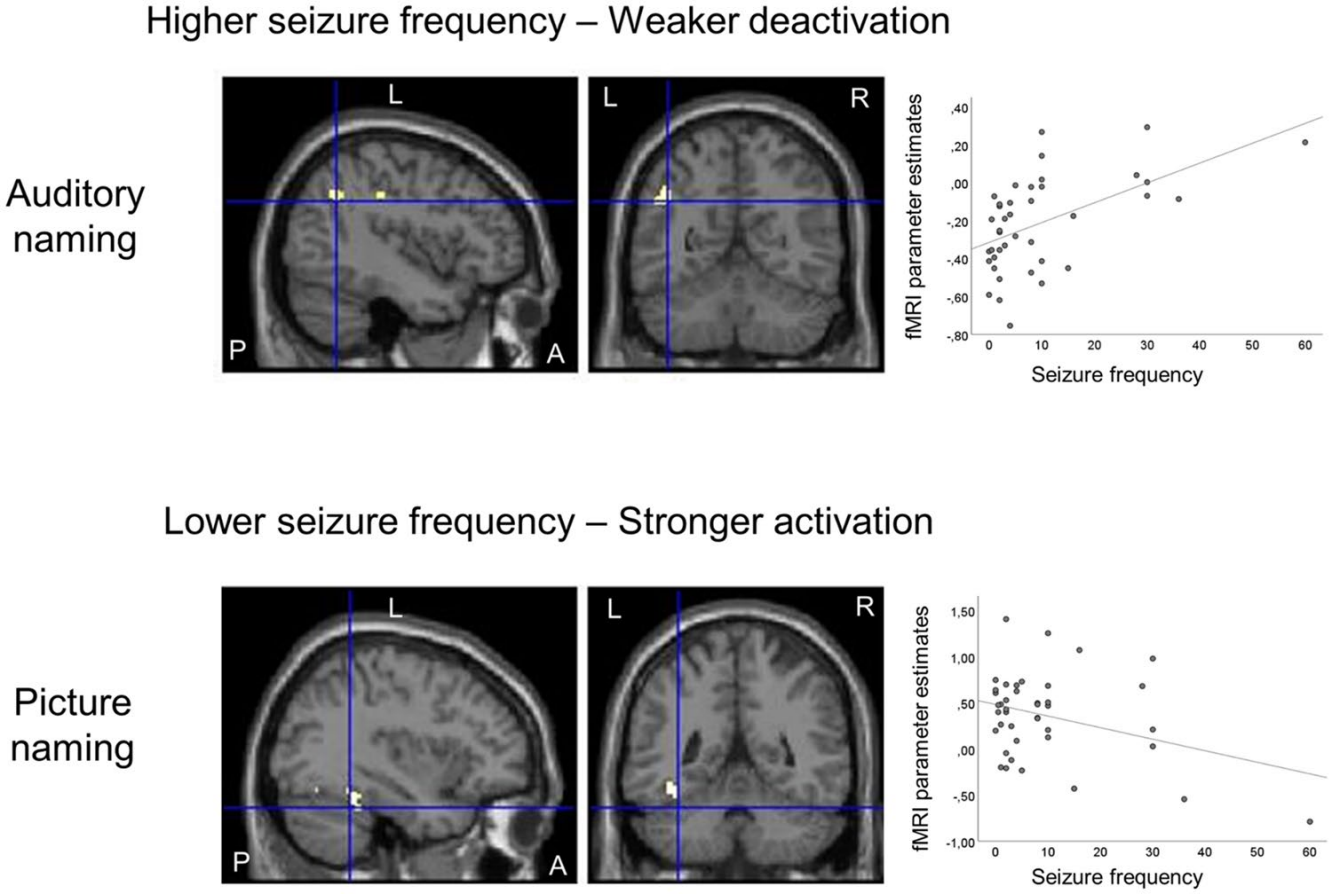
Relation of auditory naming and picture naming fMRI activations to complex partial seizure frequency in left TLE patients. Activations are shown superimposed on coronal and sagittal images at *p* < 0.001, uncorrected and the crosshair indicates the orthogonal slices. For auditory naming (upper row), higher seizure frequency was related to weaker deactivation of the left angular gyrus (crosshair) and left inferior parietal lobule. For picture naming (lower row), lower seizure frequency was associated with stronger activations in the left fusiform gyrus and left inferior occipital gyrus. Scatterplots show correlations of fMRI parameter estimate with seizure frequency. *A *anterior, *L *left, *P *posterior, *R *right, *TLE *temporal lobe epilepsy

During auditory naming, LTLE patients with higher seizure frequency showed weaker deactivations in the left angular gyrus and left inferior parietal lobule (Fig. 5; Table 6). Group comparisons indicated stronger associations in LTLE than RTLE patients, in which no significant correlations were observed.

For picture naming, LTLE patients showed an association between lower seizure frequency and stronger activations in the left fusiform gyrus and left inferior occipital gyrus (Fig. 5; Table 6). Group comparisons also confirmed stronger associations in LTLE compared to RTLE patients, in which no association was detected.

Neither patient group showed a significant association of disease characteristics with verbal fluency activations.

## Discussion

Using the recently introduced auditory and visual naming fMRI with active control conditions [5], we corroborate the relation of left posterior temporal lobe naming fMRI activations to clinical naming performance in TLE patients. The significance of left posterior lateral and medial temporal lobe regions for semantic processing is well documented [18, 19]. In line with previous findings [20], picture naming elicited activations preferentially in posterobasal temporal and temporooccipital regions associated with visual object processing, including the posterior fusiform gyrus. Auditory naming activated more lateral cortical areas including the inferior and middle temporal gyri, and more extensively involved inferior frontal and premotor regions compared to picture naming. Overall, this is consistent with the notion that auditory naming tasks are associated with increased semantic/executive control as well as working memory demands [21]. In accord with previous experiments [22, 23], auditory naming led to activations in both anterior and posterior temporal regions, while picture naming only activated the posterior part of the fusiform gyrus. We recently performed a longitudinal analysis in 46 TLE patients undergoing ATLR, comprising a subgroup of 35 patients of the cohort of this study, in which we demonstrated that posterior inferior temporal and posterior fusiform fMRI activation during auditory, respectively, picture naming can be used to predict naming decline after ATLR with very high specificity at maximum sensitivity [24].

Here, we extend on these findings by showing that naming performance not only relates to activation in discrete temporal lobe regions but also requires successful deactivation of task-negative areas. Furthermore, we demonstrate how clinical characteristics influence the organisation and reorganisation of these naming networks, particularly in patients with left TLE. These results are also in accord with our earlier findings of stronger functional connectivity of language networks seeding from the left basal temporal lobe in patients with shorter epilepsy duration, or with later age at onset of seizures [8].

In contrast to findings during auditory and picture naming fMRI, verbal fluency fMRI primarily led to frontal lobe activations, that also related to clinical naming performance, but were unaffected by TLE characteristics. This is in line with the notion that language network reorganisation in refractory TLE may predominantly affect temporal lobe locations [24], and argues in favour of the use of naming fMRI for clinical purposes, in addition to standard verbal fluency fMRI, the latter mainly activates frontal lobe regions, and is less specific to predict naming decline following temporal lobe surgery [3, 24].

During auditory naming, both LTLE and RTLE patients with a later age of epilepsy onset showed stronger left anterolateral temporal lobe activations, which relate to complex semantic processing and naming [25–28]. In contrast, an earlier age of onset was associated with reduced deactivation in task-negative regions, which suggests a “failure to deactivate” the default mode network [7, 29]. Previous studies in healthy individuals suggest a “developmental switch” from an interhemispheric language network organisation at birth to an intrahemispheric, left-lateralized pattern in adults [30, 31], and a detrimental influence of epilepsy onset on intrahemispheric segregation may account for our findings in both LTLE and RTLE patients.

Disease duration and seizure burden primarily affected auditory and picture naming fMRI activations in LTLE patients. Patients with shorter disease duration and lower seizure frequency showed stronger activations in brain regions involved in naming performance and semantic processing [25, 27, 30], including anterior lateral and posterior medial and lateral temporal lobe regions, and frontal lobe and occipital lobe regions. Longer disease duration and higher seizure frequency, conversely, was associated with reduced deactivation of task-negative regions.

Effective deactivation of task-negative areas is necessary for successful task execution [6, 7], which is concordant with our observation of better out-of-scanner naming performance in LTLE patients with a later age of onset of epilepsy, while LTLE patients overall still performed worse than RTLE patients and controls. This is in accord with our previous work, showing that functional connectivity of left temporal lobe language networks is impaired in left TLE patients with longer disease duration [8]. Our current results build on these findings, by showing that besides reorganisation of discrete language-essential regions in the temporal and frontal lobes, the language network in TLE is apparently affected on a more complex scale, including task-negative brain regions that are remote from the seizure focus. This notion is further supported by pathology studies, suggesting that not only generalised seizures but also repeated complex partial seizures may cause neuronal injury [32–34]. Such damage may contribute to impaired language performance in TLE [35], owing to the particular vulnerability of the language network to the effects of epileptic activity [36]. Further, propagation of focal seizures may trigger reorganisation not only adjacent to the focus, but also in remote ipsi- and contralateral brain areas [37, 38].

Verbal fluency activations were not affected by age of onset of epilepsy, disease duration or seizure frequency in either patient group, which is consistent with the concept that temporal lobe language networks are more susceptible to reorganization than frontal lobe networks [39].

### Strengths and limitations

We used active control conditions for our overt auditory and picture naming tasks, thus subtracting activations caused by the type of stimulus presentation (auditory/visual), as well as motor cortex activations and potential movement artifacts caused by overt speech production [5, 8]. The design of our naming tasks allowed excellent in-scanner performance in both patients and controls, while on the more challenging out-of-scanner graded naming tasks, left TLE patients performed less well than right TLE patients and controls. This replicates previous findings that TLE patients may be able to perform within the normal accuracy range on simple naming and semantic memory tasks, while impairment may manifest during tasks with increasing difficulty [40–42].

Seizure frequency was assessed from patient history and might not reflect a true measure of seizure burden in the individual subject [43]. Although drug load was comparable in LTLE and RTLE patients (median AEDs = 2 in both groups), a potential effect of medication on fMRI activations was not accounted for. There are indications that Topiramate and Zonisamide may affect language fMRI activation patterns [10, 11], however, the number of patients taking Topiramate or Zonisamide and their daily doses were comparable between TLE groups. It is important to note that 34 out of 72 patients had hippocampal sclerosis, which typically develops at an early age. This might contribute to our findings on the correlation between fMRI activation level and age at onset of epilepsy. MRI findings were heterogenous in our sample, which should be addressed in future investigations with subgroup analyses of adequate size.

### Clinical implications

Resection of the anterior temporal lobe represents an effective treatment option for patients with refractory TLE and leads to seizure remission in up to 80% of patients [44], but bears a considerable risk of post-operative naming and word-finding difficulties, which may be predicted from preoperative language fMRI. We show that functionally relevant temporal lobe fMRI activations are related to shorter disease duration, later age of onset and lower seizure frequency, whereas less favourable disease characteristics relate to impaired deactivation of task-negative regions, particularly in LTLE, which might imply a higher risk for these patients to develop naming deficits following temporal lobe resection. This is in line with our previous finding that naming fMRI activation in the ipsilateral posterior medial and lateral temporal lobe is predictive of naming decline, even when the cortical region showing naming activation is spared, and extends on these results by identifying further potential risk stratifiers from combining clinical and fMRI characteristics.

Our findings may have implications for surgical planning and stimulation language mapping, and influence the prediction and management of postoperative naming deficits, which may result in high-risk patients electing not to have surgery, or to consider less invasive surgical options such as laser interstitial thermal therapy (LITT).

## Conclusions

A younger age of onset of epilepsy affected temporal lobe language network organisation in both LTLE and RTLE patients, while frontal lobe language regions appeared spared. Later age of onset, shorter disease duration and lower seizure frequency were associated with naming fMRI activations in temporal lobe areas, which in turn were shown to relate to better clinical naming performance. Earlier age of onset, longer disease duration and higher seizure frequency were associated with a failure to deactivate task-negative regions, particularly in LTLE patients, which was reflected in impaired clinical naming performance.

These findings suggest that temporal lobe language networks are particularly vulnerable to early and repeated insults resulting from chronic epilepsy, especially in LTLE patients.

### Electronic supplementary material

Below is the link to the electronic supplementary material.

Supplementary file1 (DOCX 304 kb)

## Data Availability

Data supporting the study findings are available from the corresponding author upon reasonable request.

## Appendix

See Table 7.
