# Supplementary material for: Impaired naming performance in temporal lobe epilepsy: language fMRI responses are modulated by disease characteristics
Source: J Neurol. 2020 Aug 3;268(1):147–60. doi: 10.1007/s00415-020-10116-x (PMC7815622; doi:10.1007/s00415-020-10116-x)
Supplement: Supplementary file 1 — Supplementary file1 (DOCX 304 kb) [file 415_2020_10116_MOESM1_ESM.docx]

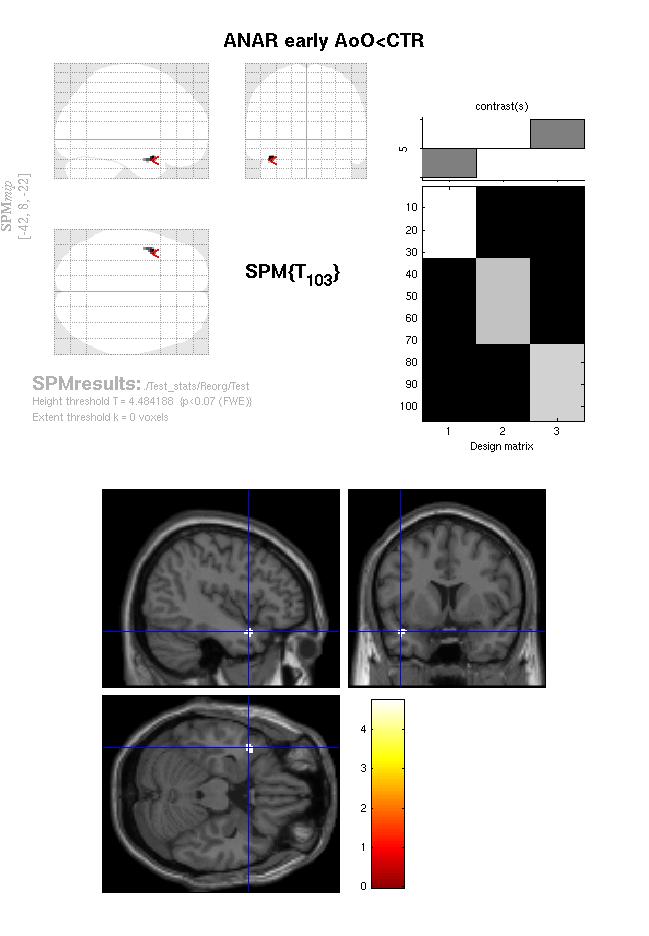


Supplementary Figure 1. Effect of age at onset of seizures on whole-brain activation during auditory naming. Patients with early onset of seizures (< age 18) show less activation in the left temporal pole (superior temporal gyrus) compared to controls (Z= 4.50; MNI coordinates -42 8 -22). Activations are shown superimposed on sagittal images at p < 0.05, corrected for multiple comparisons (FWE).

Note: FWE = family-wise error; MNI = Montreal Neurological Institute


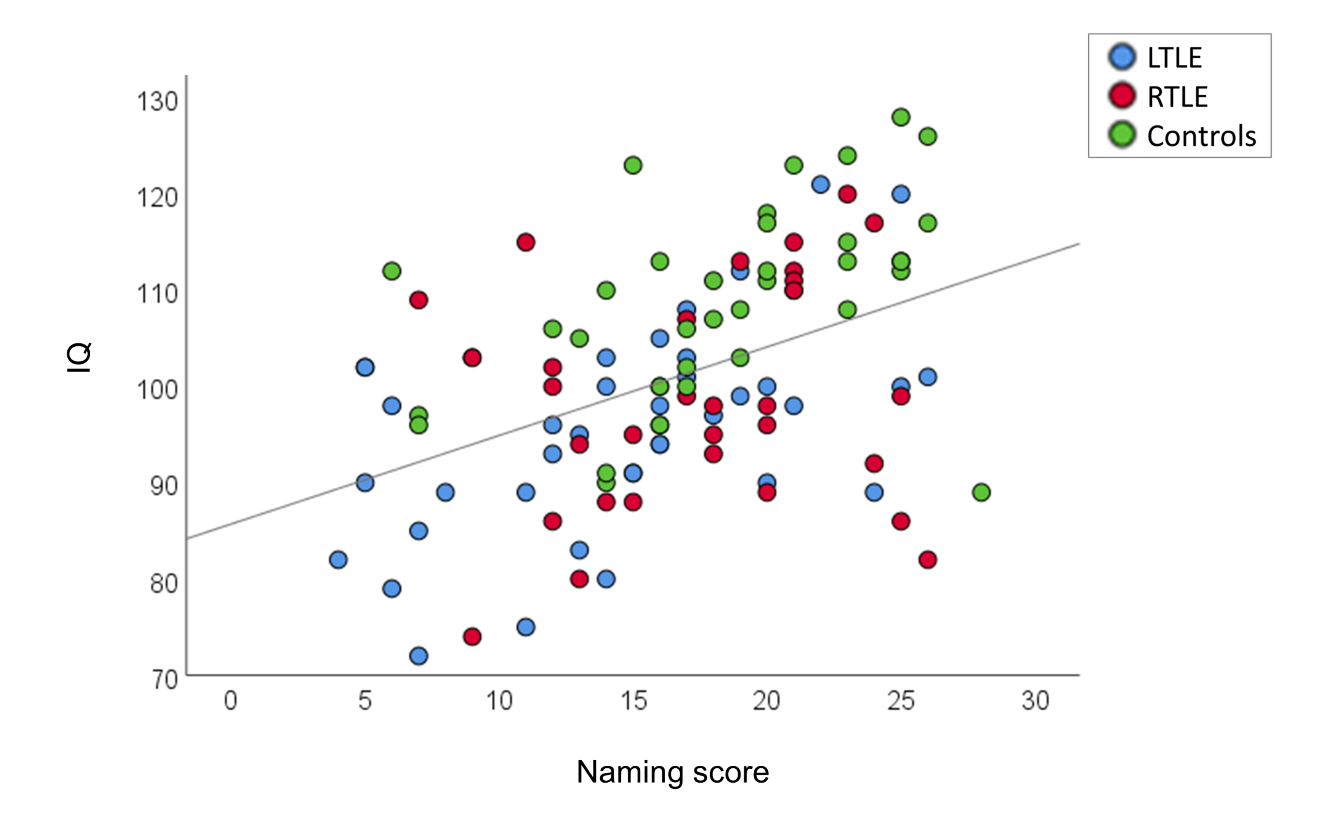


Supplementary Figure 2. Correlation of clinical naming scores with estimated verbal IQ. Across groups, higher IQ was correlated with better clinical naming scores (Pearson’s r = 0.45, p<0.001).
